# Lack of Genomic Instability in Bone Marrow Cells of SCID Mice Exposed Whole-Body to Low-Dose Radiation 

**DOI:** 10.3390/ijerph10041356

**Published:** 2013-04-02

**Authors:** Kanokporn Noy Rithidech, Chatchanok Udomtanakunchai, Louise Honikel, Elbert Whorton

**Affiliations:** 1 Pathology Department, Stony Brook University, Stony Brook, NY 11974, USA; E-Mail: lmhonikel@hotmail.com; 2 Department of Radiologic Technology, Faculty of Associated Medical Sciences, Center of Excellence for Molecular Imaging, Chiang Mai University, Chiang Mai 50200, Thailand; E-Mail: oilchat@hotmail.com; 3 Institute of Human Infections and Immunology, Galveston National Laboratory, University of Texas Medical Branch, Galveston, TX 77555, USA; E-Mail: ewhorton@utmb.edu

**Keywords:** low-dose radiation, SCID mouse, bone marrow cells, genomic instability, chromosome aberrations

## Abstract

It is clear that high-dose radiation is harmful. However, despite extensive research, assessment of potential health-risks associated with exposure to low-dose radiation (at doses below or equal to 0.1 Gy) is still challenging. Recently, we reported that 0.05 Gy of ^137^Cs gamma rays (the existing limit for radiation-exposure in the workplace) was incapable of inducing significant *in vivo* genomic instability (measured by the presence of late-occurring chromosomal damage at 6 months post-irradiation) in bone marrow (BM) cells of two mouse strains, one with constitutively high and one with intermediate levels of the repair enzyme DNA-dependent protein-kinase catalytic-subunit (DNA-PKcs). In this study, we present evidence for a lack of genomic instability in BM cells of the severely combined-immunodeficiency (SCID/J) mouse (which has an extremely low-level of DNA-PKcs activity) exposed whole-body to low-dose radiation (0.05 Gy). Together with our previous report, the data indicate that low-dose radiation (0.05 Gy) is incapable of inducing genomic instability *in vivo* (regardless of the levels of DNA-PKcs activity of the exposed mice), yet higher doses of radiation (0.1 and 1 Gy) do induce genomic instability in mice with intermediate and extremely low-levels of DNA-PKcs activity (indicating an important role of DNA-PKcs in DNA repair).

## 1. Introduction

It is known that high doses of radiation induce deleterious effects in exposed cells or tissues. However, it is unclear whether such harmful effects will be found at doses less than or equal to the existing limit for radiation exposure in the workplace, *i.e.*, less than or equal to 0.05 Gy/year of low linear energy transfer (LET) radiation (e.g., X or γ rays). Information on the capacity of low doses of low LET radiation to reduce cytogenetic damage to levels below the spontaneous rate is limited [1,2,3]. Hence, assessment of potential health risks associated with exposure to radiation at these low-dose levels is still a challenging public health issue. Reliable information about radiation-induced detrimental effects, and the reduction of uncertainties in the assessment of health risks, requires that data be obtained by using appropriate *in vivo* systems, since *in vitro* systems cannot fully mimic complex *in vivo* situations. *In vivo* radiological studies using humans are not possible. Therefore, *in vivo* animal systems are critically important surrogates for assessment of health risks from exposure to low-dose radiation. 

Recently, we evaluated the *in vivo* induction of genomic instability, expressed as late-occurring chromosome aberrations (CAs) in bone marrow (BM) cells collected at 6 mos post-irradiation from two strains of mouse with different genetic backgrounds [1]. We studied the radiosensitive BALB/cJ mouse and the radioresistant C57BL/6J mouse following a whole-body exposure to various doses of ^137^Cs γ rays (0, 0.05, 0.1, and 1.0 Gy). The induction of radiation-induced genomic instability was studied because it is a fundamental mechanism known to elevate cancer risk. We found that a single low dose of ^137^Cs γ rays (*i.e.*, 0.05 Gy) was incapable of inducing genomic instability in metaphase cells prepared from the BM of exposed mice, but this dose of ^137^Cs γ rays was capable of reducing specific types of aberrations below the spontaneous rate over time post-irradiation. However, our results showed the induction of genomic instability by a high dose (1.0 Gy) of ^137^Cs γ rays in the radiosensitive BALB/cJ mouse with an intermediate level of the endogenous repair enzyme, the DNA-dependent protein-kinase catalytic subunit (DNA-PKcs) [4], but not in the radioresistant C57BL/6J mouse with a high level of endogenous DNA-PKcs activity [4], indicating the influence of genetic background on radiation-induced genomic instability. 

Of note, the influence of genetic background on radiosensitivity has previously been observed in human and animal studies, in both *in vivo* and *in vitro* systems [5,6,7,8,9,10]. With respect to BALB/cJ and C57BL/6J mice, such differential radiosensitivity seemingly reflects differences in DNA repair capacity due to the different levels of endogenous DNA repair enzymes (*i.e.*, DNA-PKcs activity) of these two strains, as previously suggested [4,11]. It should be noted that differences in removal of damaged cells by apoptosis [12] or the cell turnover that removes damaged cells [13] may also play a role in the disparity of radiosensitivity. In this study, we are presenting evidence for a lack of genomic instability in BM cells collected at 6 mos post-irradiation from SCID/J (C.B17-Scid) mice exposed whole-body to low-dose radiation (0.05 Gy), determined by a lack of increases in the frequencies of late-occurring chromosome aberrations (CAs) in relation to those found in non-irradiated sham-control mice. In addition to late-occurring CAs, the frequencies of CAs in BM cells collected at early time-points (*i.e.*, 1 and 4 h post-irradiation) were evaluated to determine radiosensitivity in BM cells of the SCID/J mouse. Further, the frequencies of CAs in BM cells collected at 1 mo post-irradiation (reflecting karyotypic evolution after radiation exposure) were also determined.

It is well recognized that the level of DNA-PKcs activity of the SCID mouse is extremely low as a consequence of a homozygous mutation in the DNA-PKcs gene leading to deficiency in the repair of double strand breaks (DSBs) on DNA molecules [14,15,16]. Further, the SCID mouse lacks mature T and B cells due to a failure to complete V(D)J antigen receptor rejoining but is otherwise developmentally normal. The SCID mouse, however, has a high incidence of spontaneous T-cell lymphoma [17]. Since cells from SCID mice are defective in the repair of DSBs, they are known to be hypersensitive to ionizing radiation. Comparison of the LD_50 _values for whole-body irradiation [18,19] indicates that the SCID mouse is 2–3 fold more radiosensitive than its parental wild-type C.B17 mouse. It should be noted that a lack of germline mutations at tandem repeat loci has been detected in SCID mice exposed to a single dose of 1 Gy X rays, while such germline mutations were detected in the parental wild-type C.B17 mice (a substrain of the BALB/c mouse) [20]. The authors suggested that a failure of detecting such germline mutations in SCID mice is due to the high cell-killing effects of X rays on germinal cells of SCID mice in relation to their parental C.B17 mice. A higher frequency of radiation-induced unstable CAs (*i.e.*, breaks) has been observed in BM cells, fibroblasts, and spermatogonial stem cells collected from SCID mice at 24 h after exposure to various doses (ranging from 0.25 to 3 Gy) of X [21,22] or γ [23] rays, as compared to those collected from parental wild-type C.B17 mice. In contrast, a lower frequency of stable aberrations (translocations) was found in BM cells collected from SCID mice at 3 weeks following exposure to doses above 0.25 Gy, relative to those found in the parental C.B17 mouse [21,24]. The authors suggested that these findings reflect a deficiency in rejoining the broken ends of chromosomes in cells of the SCID mouse at this early time post-exposure or a loss of cells carrying aberrations incompatible with cell survival during cell divisions. Currently, there is no information on the frequency or the type of CAs in BM cells collected from SCID mice at later time-points beyond 3 weeks after exposure to radiation, in particular at the low-dose range (less or equal to 0.1 Gy). The resulting data from this study fill this knowledge gap. 

## 2. Materials and Methods

Experimental methods (except those in the next section describing animals) were the same as those recently reported by Rithidech *et al*. [1] and will therefore be briefly summarized.

### 2.1. Animals

Male SCID/J (also known as C.B17-Scid/J or CBySmn.CB17-Prkdc^scid^/J) mice were purchased from the Jackson Laboratory (Bar Harbor, ME, USA). They were 8–10 weeks old at the time of delivery and were acclimatized for two weeks prior to γ-irradiation. Due to their severe immuno-deficiency, they were kept in a maximum isolation unit throughout the study, except during irradiation, with a light cycle of 12 h light/12 h dark. Sterile food and drinking water were available to the mice *ad libitum*. Similar to the BALB/cJ and C57BL/6J mice used in our previous study [1], these male mice were not littermates, and it was important that they were housed one in a cage to prevent fighting or cannibalism. Mice were housed and cared for in a facility accredited by the American Association for Accreditation of Laboratory Animal Care (AAALAC). All animal handling procedures were performed under the guidelines approved by the Institutional Animal Care and Use Committee (IACUC) of Stony Brook University (SBU).

### 2.2. Irradiation

Four groups of 20 SCID/J mice (10–12 weeks old at exposure) were given a whole-body total dose of 0, 0.05, 0.1, or 1.0 Gy of ^137^Cs γ rays (at the dose rate of 0.75 Gy/min) using the Gamma Cell40 (Atomic Energy of Canada, Ltd, ON, Canada) located in the Division of Laboratory Animal Resources of Stony Brook University (SBU). A high dose of 1.0 Gy was used as a positive control. Mice exposed to 0 Gy of ^137^Cs γ rays served as non-irradiated sham controls. Details of dosimetry and exposure have been presented elsewhere [1]. 

### 2.3. Collection of BM Cells and Cytogenetic Assays

At each harvest time (*i.e.*, 1 h, 4 h, 1 mo, and 6 mo post-irradiation), BM cells were collected from each mouse for the analysis of CAs. The frequencies of CAs detected at 1 and 4 h post-irradiation are indicative of early responses; while those detected at 1 and 6 mo post-irradiation represent the occurrence of karyotypic progression and genomic instability, respectively. There were five mice in the non-irradiated sham control and the 1.0 Gy exposed groups. Due to death of two mice by natural causes during the 6 mo of the study, there were only four mice in the 0.05 Gy and the 1.0 Gy exposed group. We collected BM cells from each mouse by flushing both femurs and tibiae with 10 mL of McCoys’ 5A medium (Invitrogen, Grand Island, NY, USA). It is important to note that the presence or absence of radiation-induced genomic instability reported here was determined by the occurrence of late or delayed chromosomal damage detected in the progeny of BM cells of mice exposed in a study conducted with a combination of *in vivo* irradiation and *in vivo* expression of genomic instability. In contrast, previously reported data from other groups of investigators [25,26,27,28,29,30] were derived from studies conducted with a combination of either: (*i*) *in vitro* irradiation and *in vitro* expression of genomic instability, or (*ii*) *in vivo* irradiation and *in vitro* expression of genomic instability, or (*iii*) *in vitro* irradiation and *in vivo* expression of genomic instability. 

Methods for culturing and harvesting metaphase cells for cytogenetic analysis have recently been presented in Rithidech *et al.* [1]. In brief, we obtained metaphase chromosomes from BM cells harvested at 1 and 4 h post-irradiation by the addition of colcemid (0.2 µg/mL) into freshly prepared BM cultures that were incubated in a water bath at 37 °C for 2 h. The incubation time was short to ensure the accurate measurement of the type and the number of chromatid- or G2-type aberrations occurring at 1 and 4 h post-irradiation. Importantly, if the incubation time was prolonged (e.g., 24 h), the heavily damaged cells might have been lost due to their inability to survive a subsequent cell division. This phenomenon would result in obtaining imprecise frequencies of initial CAs induced by radiation. In contrast, for the measurement of late-occurring CAs (BM cells harvested at 1 and 6 mo following irradiation), a short-term (24 h) culture was used. This protocol has been a routine procedure in our laboratory [31] because it consistently provides a high yield of metaphase cells needed for the analysis of CAs, in particular the stable-type CAs and clones of aberrant cells that survive cell division. It is recognized that a subset of BM cells might have undergone a cell cycle during the 24 h incubation. Hence, a dilution of CAs (in particular loss of cells with breaks and/or unstable-type aberrations incapable of surviving cell division) may have occurred. This, in turn, may have inconsequentially changed the absolute numbers of abnormal cells or the frequencies of CAs. However, such CAs may be unrelated to the induction of genomic instability and an eventual neoplastic transformation of hematopoietic cells.

A standard cytogenetic method using freshly prepared Carnoy’s solution (3:1 vol/vol of absolute methanol/glacial acetic acid) as fixative [32] was applied to harvest metaphase BM cells. After 2–3 washings in fixative, we stored the BM cells in fixative at 4 °C until used in slide preparation for the analysis of CAs. The extent and type of initial chromosomal damage (chromatid-type or G2-aberrations) in metaphase cells prepared from BM cells collected at 1 or 4 h post-irradiation provide a measure of the sensitivity of cells to radiation. Bone marrow cells collected at 1 and 6 mo post-irradiation were used for measuring karyotypic progression and expression of genomic instability induced by radiation exposure (measured by the presence of late-occurring CAs), respectively. If genomic instability does occur, this schedule of sample collection will allow the detection of both clonal and non-clonal CAs (both chromatid- and chromosome-type aberrations) in descendants of cells from exposed mice, as we detected in our previous studies [1]. In addition, the occurrence of stable exchanges (*i.e.*, translocations) at late time-points (in particular at 6 mo post-irradiation) provide evidence of a fraction of surviving cells (carrying damage) that may be at an increased risk for subsequent neoplastic transformation. 

### 2.4. Fluorescence in situ Hybridization (FISH) Assay

**Figure 1:**
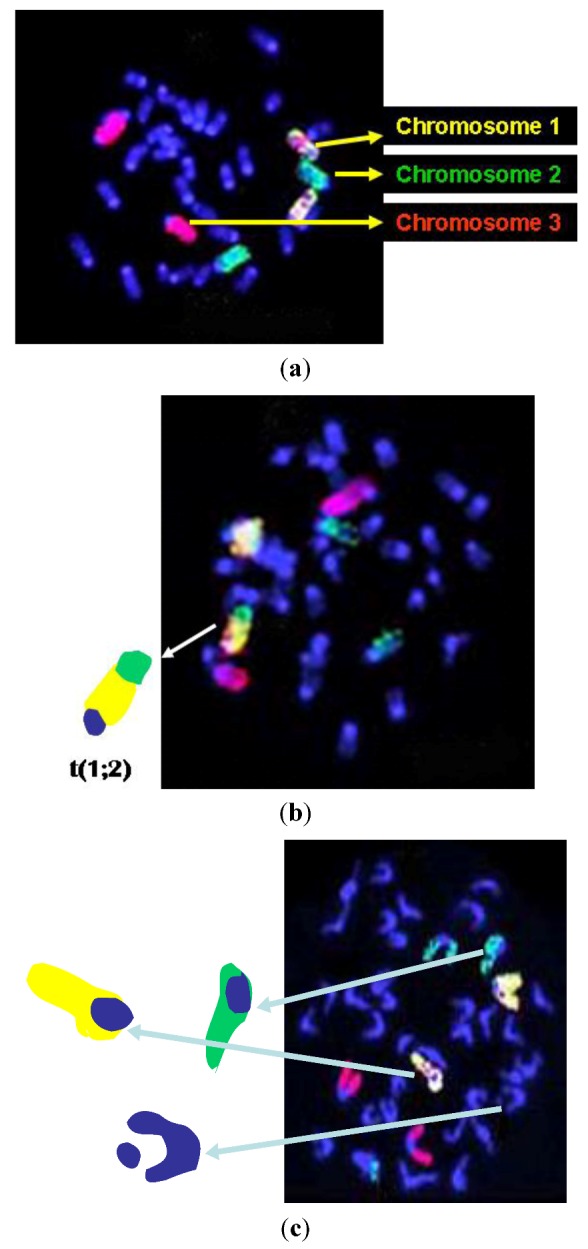
(**a**) Representative image of a normal metaphase cell; (**b**) Representative image of a metaphase with a translocation between mouse chromosomes 1 and 2 (arrow), and (**c**) Representative image of a metaphase with chromatid breaks on chromosomes 1, 2, and nP.

Each slide was stained (“painted”) simultaneously with concentrated paint probes for chromosomes 1, 2, and 3 (purchased from Vysis/Cambio, Inc., Cambridge, UK), using the same procedure as previously reported [1]. Briefly, the probe for chromosome 1 was labeled with biotin- and fluorescein-isothiocyanate (FITC); while, the probe for chromosome 2 was labeled with FITC and the probe for chromosome 3 was labeled with biotin. All other metaphase chromosomes were counterstained blue with Vectashield anti-fade, containing 400 ng/mL 4',6-diamidino-2-phenylindole (DAPI; Vector Laboratories, Burlingame, CA, USA). Using this protocol, chromosome 1 appeared yellow (or a speckled mixture of red/green), chromosome 2 appeared green, chromosome 3 appeared red, and all other chromosomes (non-painted, referred to as nP in the Tables) were blue (as shown in Figure 1(a–c). Metaphase images were captured and stored using a digital imaging ISIS system (MetaSystems, Inc., Waltham, MA, USA) with a cooled CCD camera equipped with a special FISH software ISIS program. Of note, we have two specific reasons for choosing mouse chromosomes 1, 2, and 3 for the analysis of CAs by means of FISH. First, existing databases indicate the *in vivo* persistence of damage to these chromosomes following exposure to high doses of low LET radiation [33,34,35,36,37,38], and second, these chromosomes are the largest chromosomes in the mouse.

### 2.5. Chromosome Aberration Scoring

All slides were coded so that the scorer had no knowledge of the treatment group of the mice from which the slides were prepared. The code remained unknown until the data were analyzed. The scoring of aberrations involving each of the individual painted metaphase chromosomes was done by using the criteria previously suggested [39], which had been presented in detail in our recent work [1]. All other chromatid- or chromosome-type aberrations and gross structural abnormalities involving nP chromosomes were determined simultaneously in the same metaphase cells that were used for scoring CAs involving painted chromosomes. We also recorded the number of cells with CAs (abnormal cells) for each treatment group. Of note, gaps (those with a discontinuity shorter than the chromatid width or non-displacement) were recorded separately. At late time-points, the criterion for determining a clone of cells suggested previously [40] was used, *i.e.*, two or more cells with the same structural abnormalities on the same chromosomes in each individual mouse.

### 2.6. Statistical Analysis

The same approach used in our recent work with BALB/cJ and C57BL/6 mice [1] was applied, Briefly, the average square root transformation (ASQRT, √X + √(X + 1) where X is the observed frequency of each type of aberration) was applied to each animal’s aberration frequency to achieve reasonable normality and reasonably homogeneous inter-animal variability within treatment-combination groups [41]. The analysis of variance (ANOVA) methods appropriate for two-factor factorial experiments were used to evaluate the resulting chromosome data for the main or overall effects of time, radiation dose, and their interaction. One factor was radiation dose-level, and the other was time post-exposure. A *p* value of <0.05 was considered statistically significant.

## 3. Results and Discussion

There were many heavily damaged cells (those containing at least 10 breaks, and also known as pulverized cells) in BM cells collected from SCID/J mice exposed to 1.0 Gy of ^137^Cs γ rays at 1 and 4 h post-irradiation. Such pulverized cells were infrequently found in BM cells from the 0.05 Gy and the 0.1 Gy exposed groups, while none was observed in the non-irradiated sham controls. These heavily damaged cells are incompatible with cell survival so they are highly likely to be unable to survive cell division, making them unlikely to be representative of cells at risk for late health risks (such as cancer induction). Hence, we recorded the frequencies of pulverized cells, but we neither include them in statistical analyses nor show them in tables and figures.

### 3.1. Early Time-Points (1 and 4 h Post-Irradiation)

**Figure 2:**
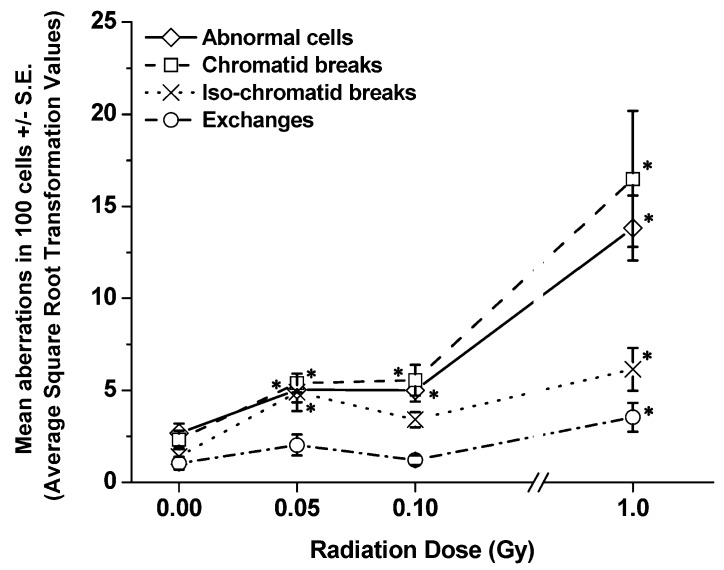
Frequencies of each type of aberration per 100 cells scored (±S.E.) detected in BM cells collected from SCID/J mice at 1 h post-irradiation. Significant differences in the frequencies of each type of CA in BM cells of exposed mice, as compared to the frequencies detected in non-irradiated sham controls are shown at “**∗**”.

**Figure 3:**
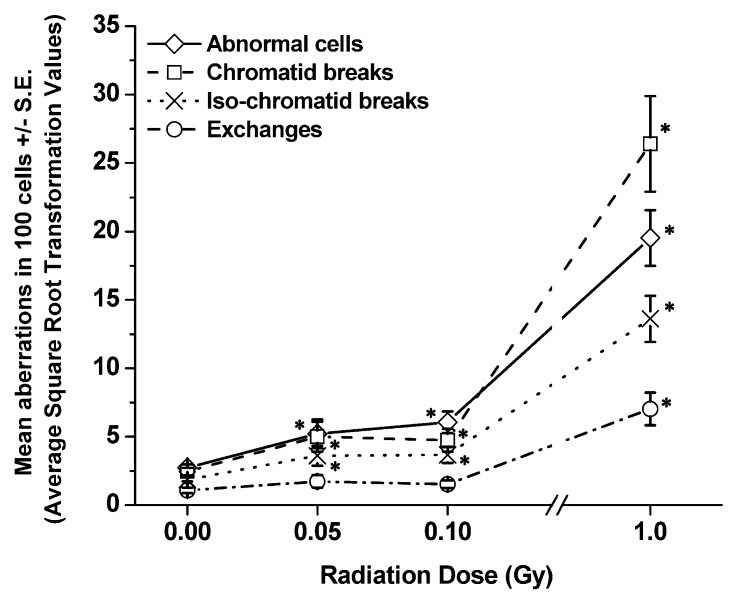
Frequencies of each type of aberration per 100 cells scored (±S.E.) detected in BM cells collected from SCID/J mice at 4 h post-irradiation. Significant differences in the frequencies of each type of CA in BM cells of exposed mice, as compared to the frequencies detected in non-irradiated sham controls are shown at “**∗**”.

Table 1 and Table 2 show the details of pooled raw data for each aberration type (*i.e.*, abnormal cells, breaks, and exchanges) and the chromosome(s), both painted (chromosomes 1, 2 and 3) and non-painted (nP) ones, involved in every aberration type determined at 1 and 4 h, respectively, from each group of exposed SCID/J mice. The total number of cells scored for each treatment group was also shown in each table. Figure 2 and Figure 3 present the frequencies of each type of chromatid (G2) aberration per 100 cells scored (± S.E.), including abnormal cells, detected in BM cells collected from SCID/J mice at 1 and 4 h post-irradiation, respectively. The numbers presented on the graphs were the ASQRT numbers (Table 3 and Table 4), which also were used for evaluating statistical significance (see Section 2, Materials and Method). It also should be noted that the background frequencies of CAs in BM cells of SCID mice used in our study were similar to those previously reported [21,22,24].

**Table 1:** Cytogenetic data from bone marrow cells collected at 1 h after exposure of male SCID/J mice to varying low doses of ^137^Cs γ rays, including a high dose of 1.0 Gy serving as a reference dose. All aberrations are chromatid-types.

	Total	Total	Total	Chromatid breaks				Total	Iso-chromatid breaks				Total	Exchanges
Dose	number of	number of	number of	(Chromosome involved)				number of	(Chromosome involved)				number of	
(Gy)	cells	abnormal	chromatid	Chromosome				Iso-chormatid	Chromosome				exchanges	
	scored	cells	breaks	(1)	(2)	(3)	(nP)	breaks	(1)	(2)	(3)	(nP)		
**0**	**1,078**	**44**	**34**	2	3	1	28	**13**	3	2	2	6	**5**	**t(nP;1),t(nP;1),t(nP;nP),t(nP;3), ins(nP;1;nP)**
**0.05**	**483**	**37**	**39**	3	4	0	49	**27**	6	9	5	7	**3**	**recipt** **(nP;3),t(np:1); t(nP;1)**
**0.10**	**908**	**110**	**133**	14	8	6	95	**53**	14	8	9	22	**8**	**t(nP;1),t(nP;3),t(2;nP),t(nP;1), t(nP;2),t(nP;3),dic(3;nP), dic(nP;nP)**
**1.00**	**513**	**311**	**475**	35	22	30	388	**62**	13	10	15	24	**18**	**t(1;2) two cells,t(2;3),t(nP;1)t(nP;1), t(3;2) two cells,t(3;nP), recip t(3;nP), t(nP;1),t(nP;1),t(nP;1),t(nP;2), t(nP;2),t(nP;3),t(nP;3),t(nP;3), dic(nP;nP)**

*t* Translocation (incomplete type), *recip t* Reciprocal translocation, *dic* Dicentric, *ins* Insertion, *nP* Non-painted Chromosome.

**Table 2:** Cytogenetic data from bone marrow cells collected at 4 h after exposure of male SCID/J mice to varying low doses of ^137^Cs γ rays, including a high dose of 1.0 Gy serving as a reference dose. All aberrations are chromatid-types. *t* Translocation (incomplete chromatid-type), *RT* Robertsonian translocation, *dic* Dicentric, *ins* Insertion, *nP* Non-painted chromosome.

	Total	Total	Total	Chromatid breaks				Total	Iso-chromatid breaks				Total	Exchanges
Dose	number of	number of	number of	(Chromosome involved)				number of	(Chromosome involved)				number of	
(Gy)	cells	abnormal	chromatid	Chromosome				Iso-chromatid	Chromosome				exchanges	
	scored	cells	breaks	(1)	(2)	(3)	(nP)	breaks	(1)	(2)	(3)	(nP)		
**0**	**1,056**	**38**	**28**	5	2	1	20	**20**	8	1	1	9	**7**	**t(nP;1),t(nP;2),t(nP;1),t(nP;1), ins(1;nP;1),ins(3;1) two cells**
**0.05**	**383**	**19**	**18**	3	2	1	12	**8**	1	1	0	6	**1**	**t(nP;1)**
**0.10**	**935**	**125**	**81**	11	5	7	58	**48**	17	4	7	20	**11**	**t(1;3),t(nP;3),t(nP;1),t(nP;3), ring(nP), dic(nP;nP), dic(nP;nP),dic (nP;nP), ins(nP;1;nP),RT(1;2),RT(nP;nP)**
**1.00**	**256**	**137**	**242**	25	11	13	193	**73**	16	8	11	38	**14**	**t(2;1),t(nP;1),t(nP;2),t(nP;2),t(nP;2),recip-t(nP;nP),t(3;1)** **, t(nP;1),t(nP;2),t(nP;3), ins(nP;2;nP),ins(nP;3;nP), ins(nP;3;nP),RT(nP;nP)**

**Table 3:** Average square root transformation values (√X + √(X+1)) of mean aberrations in 100 cells scored ± standard error of the mean (S.E.) derived from the raw data of the frequencies of each type of aberration, including abnormal cells as shown in Table 1 (1 h post-irradiation).

Dose (Gy)	Total number of abnormal cells ± S.E.	Total number of chromatid breaks ± S.E.	Total number of iso-chromatid breaks ± S.E.	Total number of exchanges ± S.E.
0	2.67 *±* 0.52	2.33 ± 0.47	1.40 ± 0.41	1.04 ± 0.35
0.05	5.03 ± 0.68	5.40 ± 0.51	4.90 ± 1.02	2.03 ± 0.57
0.10	5.01 ± 0.61	5.55 ± 0.84	3.41 ± 0.41	1.21 ± 0.25
1.00	13.82 ± 1.76	16.49 ± 3.70	6.14 ± 1.16	3.54 ± 0.79

**Table 4:** Average square root transformation values (√X + √(X+1)) of mean aberrations in 100 cells scored ± standard error of the mean (S.E.) derived from the raw data of the frequencies of each type of aberration, including abnormal cells as shown in Table 2 (4 h post-irradiation).

Dose (Gy)	Total number of abnormal cells ± S.E.	Total number of chromatid breaks ± S.E.	Total number of iso-chromatid breaks ± S.E.	Total number of exchanges ± S.E.
0	2.73 *±* 0.25	2.44 ± 0.28	1.87 ± 0.15	1.07 ± 0.20
0.05	5.21 ± 1.05	4.99 ± 1.10	3.61 ± 0.73	1.73 ± 0.46
0.10	6.05 ± 0.79	4.73 ± 0.83	3.67 ± 0.59	1.52 ± 0.28
1.00	19.53 ± 2.03	26.40 ± 3.48	13.62 ± 1.69	7.02 ± 1.20

The majority of aberrations were chromatid breaks, with or without the presence of acentric fragments. Iso-chromatid breaks were also found. Chromatid exchanges included translocations (Robertsonian or centric fusion, reciprocal, and incomplete types), dicentrics, and insertions. Most of the translocations were of the incomplete one-way type because fragments were missing, regardless of radiation dose. The types of CAs were similar to those reported previously in an *in vivo* study of low-dose γ-irradiated BALB/cJ and C57BL/6J mice [1], or γ- or ^56^Fe-ion-irradiated CBA/CaJ mice [31], or those observed in *in vitro* ^56^Fe-ion-irradiated human lymphocytes [42]. These translocations occurred between either two of the painted chromosomes or between one of the painted chromosomes and a nP chromosome. There was no indication of the non-random involvement of specific chromosomes (either painted or nP) in any particular type of aberration detected at these early time-points. Dicentrics, insertions, and Robertsonian translocations (RT) were rarely found. 

At 1 h post-irradiation (Figure 2, Table 1 and Table 3), the data showed significant increases (*p values* ranging from 0.01 to 0.0004) in the frequencies of abnormal cells and chromatid breaks in BM cells collected from all exposed SCID/J mice in relation to those detected in BM cells of non-irradiated sham controls, regardless of radiation dose. Further, the frequencies of iso-chromatid breaks and chromatid exchanges were also highly significant in BM cells collected from SCID/J mice exposed to 1 Gy of ^137^Cs γ rays, related to those found in BM cells of non-irradiated sham controls. In contrast, there were no differences between the frequencies of chromatid exchanges in the BM of SCID/J mice exposed to either 0.05 or 0.1 Gy of ^137^Cs γ rays and those detected in the BM of non-irradiated sham controls. There was an apparent increase in the frequencies of iso-chromatid breaks in BM cells of SCID/J mice exposed to either 0.05 or 0.1 Gy of ^137^Cs γ rays, in relation to that detected in non-irradiated sham controls. However, a significant difference was found only in BM cells collected from mice exposed to 0.05 Gy of ^137^Cs γ rays. 

Similarly, at 4 h post-irradiation (Figure 3, Table 2 and Table 4), significant increases in the frequencies of all chromatid-type aberrations were found in BM cells collected from SCID/J mice exposed to 1 Gy of ^137^Cs γ rays (*p* values ranging from 0.0001 to 0.0005), related to those found in their non-irradiated sham controls. For the 0.05 Gy and the 0.1 Gy exposed mice, all types of aberrations, except (chromatid) exchanges, were significantly higher than those found in the non-irradiated sham controls. Of note, the results indicated that the frequencies of chromatid breaks were higher than the frequencies of abnormal cells in BM cells collected from SCID/J mice exposed to 1.0 Gy of ^137^Cs γ rays at both 1 and 4 h post-irradiation, suggesting that this high dose of radiation induced more than one break (or one aberration-type) per cell. Additionally, at 1 and 4 h post-irradiation, significantly high frequencies of all chromatid-type aberrations were observed in BM cells of SCID/J mice exposed to 1.0 Gy of ^137^Cs γ rays, related to those exposed to 0.05 Gy or 0.1 Gy of ^137^Cs γ rays.

### 3.2. Late Time-Points (1 and 6 mo Post-Irradiation)

**Figure 4:**
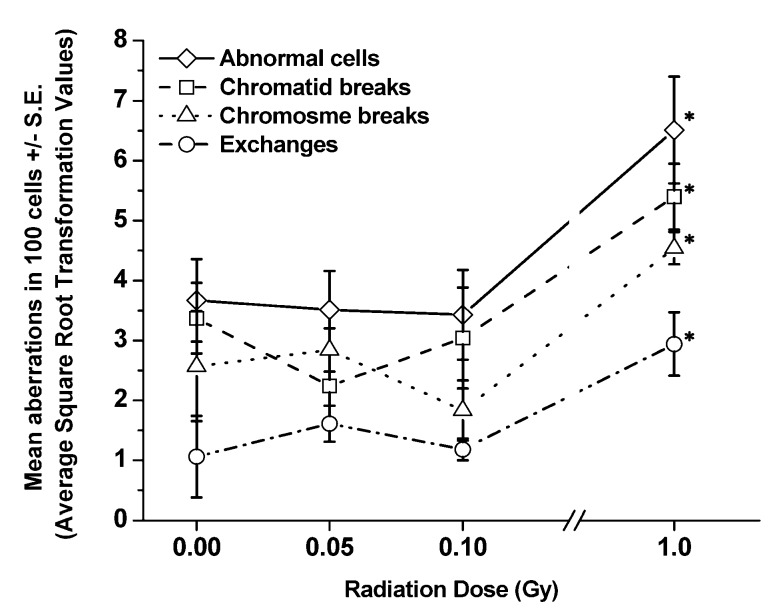
Frequencies of each type of aberration per 100 cells scored (±S.E.) detected in BM cells collected from SCID/J mice at 1 mo post-irradiation. Significant differences in the frequencies of each type of CA in BM cells of exposed mice, as compared to the frequencies detected in non-irradiated sham controls are shown at “**∗**”.

**Figure 5:**
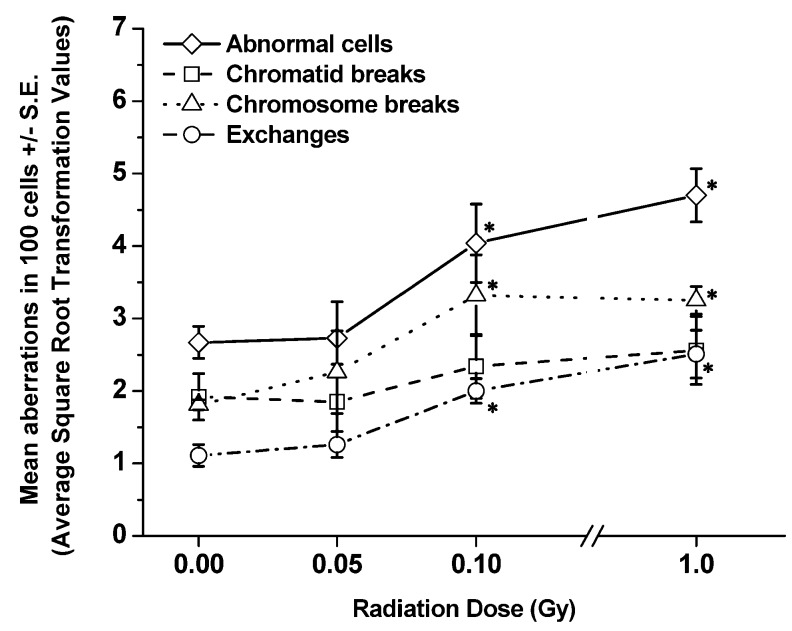
Frequencies of each type of aberration per 100 cells scored (±S.E.) detected in BM cells collected from SCID/J mice at 6 mo post-irradiation. Significant differences in the frequencies of each type of CA in BM cells of exposed mice, as compared to the frequencies detected in non-irradiated sham controls are shown at “**∗**”.

Table 5 and Table 6 show the details of pooled raw data for each aberration type (*i.e.*, abnormal cells, breaks, and exchanges) and the chromosome(s), both painted (chromosomes 1, 2 and 3) and nP ones, involved in every aberration type determined at 1 and 6 mo, respectively, from each group of exposed SCID/J mice. Figure 4 and Figure 5 present the frequencies (ASQRT values shown in Table 7 and Table 8) of each type of CA, including abnormal cells, per 100 cells scored (±S.E.), in BM cells of SCID/J mice collected at 1 and 6 mo post-irradiation, respectively. The types of CAs were similar to those observed in BM cells collected 1 or 4 h post-irradiation. Evidently, there were significant decreases in the frequencies of abnormal cells and CAs in BM cells collected from exposed SCID/J mice at both 1 and 6 mo post-irradiation, in relation to those observed in BM cells collected at early time-points. Such reductions were more pronounced in BM cells of SCID/J mice exposed to 1.0 Gy of ^137^Cs γ rays. However, the residual levels of persistent CAs (chromosome breaks, chromatid and chromosome exchanges, but not chromatid breaks) clearly remained significantly elevated, *p <* 0.05, (in relation to the levels in the non-irradiated sham controls) at 6 mo in BM cells collected from SCID/J mice exposed to 0.1 or 1.0 Gy of ^137^Cs γ rays. These findings indicate the induction of genomic instability in BM cells collected at 6 mos post-irradiation from SCID/J mice exposed to 0.1 or 1 Gy of ^137^Cs γ rays.

In contrast, there were no significant increases in the frequencies of CAs (or abnormal cells) in BM cells collected from SCID/J mice exposed to 0.05 of ^137^Cs γ rays at either 1 or at 6 mo post-irradiation. More importantly, there appeared to be a reduction (though not statistically significant, *p =* 0.1) in the frequencies of chromatid breaks in BM cells of SCID/J mice exposed to 0.05 Gy of ^137^Cs γ rays (Figure 4, filled arrow) in relation to corresponding non-irradiated sham controls. Of note, the frequencies of chromosome breaks also appeared to be reduced (but this was not statistically significant, *p =* 0.2) in BM cells of SCID/J mice exposed to 0.1 Gy of ^137^Cs γ rays (Figure 4, open-arrow). These reductions are similar to those observed in BM cells of exposed BALB/cJ mice, as previously reported [1], although the extent of reduction was less in SCID/J mice.

The majority of abnormal cells found in this study contained non-clonal aberrations (chromatid or chromosome breaks). The finding of non-clonal aberrations was similar to that previously reported in clonal populations derived from single progenitor cells by several groups of investigators [25,26,27,28,29,30]. It also should be emphasized that we determined the presence of *in vivo* genomic instability by the occurrence of late or delayed chromosomal damage in the progenies of the total population of BM cells of exposed mice from a study conducted with a combination of *in vivo* irradiation/*in vivo* expression of genomic instability. This approach was also used in our previous studies on radiation-induced genomic instability using BALB/cJ and C57BL/6J mice [1]. It should be noted that the use of a combination of *in vivo* irradiation/*in vivo* expression of genomic instability to study radiation-induced *in vivo* genomic instability is limited. Recently, this approach was used to determine the induction of genomic instability (assessed by the induction of late-occurring gene expression and microsatellite mutations) after exposure of *Caenorhabditis elegans* to 0, 0.1, 1.0, 3.0, or 10.0 Gy of X rays [43].

**Table 5:** Cytogenetic data from bone marrow cells collected at 1 mo after exposure of male SCID/J mice to varying low doses of ^137^Cs γ rays, including a high dose of 1.0 Gy serving as a reference dose. Both chromatid- and chromosome-type aberrations were detected.

	Total	Total	Total	Chromatid breaks				Total	Chromosome breaks				Total	Exchanges
Dose	number of	number of	number of	(Chromosome involved)				number of	(Chromosome involved)				number of	(Both chromatid- and chromosome-types)
(Gy)	cells	abnormal	chromatid	Chromosome				Chromosome	Chromosome				exchanges	
	scored	cells	breaks	(1)	(2)	(3)	(nP)	breaks	(1)	(2)	(3)	(nP)		
**0**	**941**	**37**	**30**	3	0	0	27	**16**	4	0	0	12	**4**	**t(nP;1),t(nP;1),t(3;nP),t(nP;nP)**
**0.05**	**393**	**10**	**4**	1	0	1	2	**5**	0	0	0	5	**1**	t*(nP;1)
**0.10**	**1,373**	**78**	**57**	8	1	8	40	**18**	7	2	2	7	**12**	**t(1;3),recip t(1;nP),t(nP;1), t(nP;1),t(nP;1),t(nP;1), ** **recip** ** t(nP;2),t(nP;nP), dic(nP;nP),dic(nP;nP),ring(nP), RT(3;nP)**
**1.00**	**518**	**49**	**27**	7 **^aa^**	4	1	15	**25**	3^a^	2	1	19	**7**	**recip** t(1;nP),t(2;nP),t(nP;1), t(nP;2),t(nP;2),t*(2;3), RT(1;nP)

*t* Translocation (incomplete, chromosome-type), t* Translocation (incomplete chromatid-type), *recip t* Reciprocal translocation, *RT* Robertsonian translocation, *dic* Dicentric, *ins* Insertion, *nP* non-painted chromosome, **^a^** clones of cells with a specific type of aberration occurring on a specific chromosome found in one mouse; **^aa^** clones of cells with a specific type of aberration occurring on a specific chromosome found in two mice.

**Table 6:** Cytogenetic data from bone marrow cells collected at 6 mo after exposure of male SCID/J mice to varying low doses of ^137^Cs γ rays, including a high dose of 1.0 Gy serving as a reference dose. Both chromatid- type and chromosome-type aberrations were detected.

Dose (Gy)	Total number of cells scored	Total number of abnormal cells	Total number of chromatid breaks	Chromatid breaks (Chromosome involved) Chromosome				Total number of Chromosome breaks	Chromosome breaks (Chromosome involved) Chromosome				Total number of exchanges	Exchanges (Both chromatid- and chromosome-types)
				(1)	(2)	(3)	(nP)		(1)	(2)	(3)	(nP)		
**0**	**1,693**	**95**	**43**	10	2	1	30	**30**	7	0	5	18	17	**t(1;2),t(1;nP),t(1;nP),recip t(2;3),t(3;nP),t(nP;1),t(nP;1), t(nP;nP), t(nP;1),t(nP;1),t(nP;1),t(nP;1),t(nP;1),t(nP;2), dic(nP;1),ins(nP;1),RT(nP;nP)**
**0.05**	**948**	**32**	**19**	2	4	2	11	**21**	5	7	1	8	8	t(nP;1),t(nP;1),t(nP;1),t(nP;1),t(nP;2), t(nP;2),t(nP;2), t*(nP;2)
**0.10**	**1,030**	**93**	**22**	7^a^	9^a^	3	3	**63**	25^aa^	4	7	27	23	t(1;nP),recip t(1;nP),t(nP;1),t(nP;1),t(nP;1),t(nP;1),t(nP;1), t(nP;1),t(nP;2),t(nP;2),t(nP;nP),t*(2;nP),t*(3;1),t*(nP;1), t*(nP;1),t*(nP;1),t*(nP;1),t*(nP;1),t*(nP;2),ins(nP;1), ins(nP;1), RT(nP;nP),RT(nP;nP)
**1.00**	**1,695**	**306**	**103**	25 ^aa^	13 ^aa^	9 ^a^	56	**153**	52 ^aa^	14 ^a^	7	80	91	**t(1;2)^a^,t(1;3)^a^,t(1;nP),recip t(1;nP), recip t(1;nP),recip t(1;nP),recip t(1;nP), recip t(1;nP),t(2;1),t(2;nP),t(2;nP), recip t(2;nP),recip t(2;nP),** **recip** t(2;nP),t(3;1),t(nP;1),t(nP;1), t(nP;1),t(nP;1),t(nP;1),t(nP;1),t(nP;1),t(nP;1),t(nP;1),t(nP;1), t(nP;1),t(nP;1),t(nP;1),recip t(nP;1),recip t(nP;1),t(nP;2), t(nP;2),t(nP;2),t(nP;2),t(nP;2),t(nP;2),t(nP;2),t(nP;2),t(nP;2), t(nP;2),t(nP;2), recip-t(nP;2),t(nP;3),t(nP;3),t(nP;3),t(nP;3), t(nP;3),recip-t(nP;3),t(nP1),t(nP1),t(nP1),t(nP1),t(nP1), t(nP1),t(nP1),t*(nP;1),t*(nP;1),t*(nP;1),t*(nP;1),t*(nP;1), t*(nP;1),t*(nP;1),t*(nP;2),t*(nP;2),t*(nP;2),t*(nP;2),t*(nP;3), t*(nP;3),t*(nP;3),ins(1;nP),ins(2;1),ins*(2;1),ins(2;3), ins(2;nP),ins(3;nP),ins(nP;1),ins(nP;1),ins(nP;1),ins(nP;1), ins(nP;1),ins(nP;2),ins(nP;2),ins(nP;2),ins(nP;2),ins(nP;3), ins(nP;3),ins(nP;3),ins(nP;3),RT(nP;nP),RT(nP;nP)

*t* Translocation (incomplete, chromosome-type), t* Translocation (incomplete chromatid-type, *recip t* Reciprocal translocation, *RT* Robertsonian translocation, *dic* Dicentric, *ins* Insertion, *nP* non-painted chromosome; ***^a^*** clones of cells with a specific type of aberration occurring on a specific chromosome found in one mouse; **^aa^** clones of cells with a specific type of aberration occurring on a specific chromosome found in two mice.

**Table 7:** Average square root transformation values [√X + √(X+1)] of mean aberrations in 100 cells scored ± standard error of the mean (S.E.) derived from the raw data of the frequencies of each type of aberration, including abnormal cells as shown in Table 5 (1 mo post- irradiation).

Dose (Gy)	Total number of abnormal cells ± S.E.	Total number of chromatid breaks ± S.E.	Total number of chromosome breaks ± S.E.	Total number of exchanges ± S.E.
0	3.67 *±* 0.69	3.37 ± 0.75	2.57 ± 0.92	1.06 ± 0.68
0.05	3.51 ± 0.68	2.24 ± 0.59	2.84 ± 0.36	1.61 ± 0.30
0.10	3.43 ± 0.75	3.04 ± 0.84	1.83 ± 0.50	1.18 ± 0.18
1.00	6.51 ± 0.89	5.40 ± 0.55	4.54 ± 0.03	2.94 ± 0.53

**Table 8:** Average square root transformation values (√X + √(X+1)) of mean aberrations in 100 cells scored ± standard error of the mean (S.E.) derived from the raw data of the frequencies of each type of aberration, including abnormal cells as shown in Table 6 (6 mo post-irradiation).

Dose(Gy)	Total number of abnormalcells ± S.E.	Total numberof chromatidbreaks ± S.E.	Total numberof chromosome breaks ± S.E.	Total number of exchanges ± S.E.
0	2.67 *±* 0.22	1.92 ± 0.32	1.81 ± 0.07	1.11 ± 0.15
0.05	2.73 ± 0.50	1.85 ± 0.52	2.26 ± 0.57	1.26 ± 0.18
0.10	4.04 ± 0.54	2.34 ± 0.44	3.32 ± 0.56	2.00 ± 0.17
1.00	4.70 ± 0.37	2.56 ± 0.47	3.25 ± 0.19	2.51 ± 0.33

With respect to the clones of cells with a specific type of aberration at late time-points, as noted in the Materials and Methods section, we used a criterion previously suggested by Rowley and Potter [40] to define a clone of cells, *i.e.*, two or more cells with the same structural abnormalities on the same chromosomes in each individual mouse. In our study, the majority of clones of abnormal cells were detected in BM cells collected at 6 mo post-irradiation from mice exposed to 1.0 Gy of ^137^Cs γ rays. These include clones of abnormal cells carrying chromatid breaks in all three painted chromosomes and cells carrying chromosome breaks in chromosomes 1 and 2. 

However, such clonalities were found in only one or two of the five mice in this exposed group (as indicated in Table 6). In addition to clones of cells with chromatid or chromosome breaks, clones of cells with clonal aberrations of rearrangement (*i.e.*, t(1;2) or t(1;3)) were found in two mice of this exposed group. 

It has been well documented that CAs are the best-characterized end point of radiation-induced genomic instability [44,45]. Numerous studies have reported the existence of genomic instability, as determined by the presence of high frequencies of late-occurring CAs in the descendants of cells surviving radiation exposure (at moderate to high dose levels of both low and high LET), in relation to those found in non-irradiated sham controls [25,26,27,29,46,47,48,49,50]. It also is well known that there are two types of chromosome instability associated with radiation-induced genomic instability, *i.e.*, non-clonal aberrations (such as chromatid breaks) and clonal aberrations (such as rearrangements) [45,46,51]. High frequencies of these two types of CAs were observed in BM cells collected from SCID/J mice exposed to 0.1 or 1.0 (but not 0.05) Gy of ^137^Cs γ rays, in relation to those found in non-irradiated sham controls. Hence, our data demonstrate that a single dose of 0.05 Gy of ^137^Cs γ rays is incapable of inducing genomic instability in BM cells collected at 6 mo post-irradiation from exposed SCID/J mice. 

There is a wide range of human populations at risk for exposure to radiation at varying doses and in many different ways, making radiation exposure a major public health issue. Such populations include individuals exposed to either intentional sources (e.g., patients who receive low levels of radiation for medical diagnosis, patients who receive high doses of radiation from radiation therapy, astronauts exposed to radiation in space, and potential victims of nuclear terrorism) or accidental sources (e.g., workers in the nuclear power industry or people living in homes surrounding nuclear power plants). Further, there is unavoidable exposure to low-level background radiation ubiquitously existing in the environment. Additionally, there is not only an increased use of low-dose radiation in daily life (e.g., for medical diagnosis or airport safety) but also an increasing concern about the possibility of radiological terrorism and/or a nuclear accident. Hence, it is important to improve our knowledge of the biological effects of low doses of radiation in order to advance the field of risk-assessment of exposure to low-dose radiation, which is still a challenging public health issue.

Our data present evidence of no induction of genomic instability, determined by the absence of increases in the frequencies of late-occurring CAs in BM cells collected at 6 mo after exposure of SCID/J mice to 0.05 Gy of ^137^Cs γ rays (the existing limit for radiation exposure in the workplace). This information is important because genomic instability is known to be a fundamental mechanism for increased cancer risk. The data also demonstrate that this low dose of low LET radiation appears to result in a reduction of specific aberration types below the spontaneous rate with time post-irradiation. Importantly, this phenomenon is similar to our previous findings in BM cells of BALB/cJ and C57BL/6J mice exposed to 0.05 Gy of ^137^Cs γ rays [1]. 

As mentioned earlier, the levels of activity of DNA-PKcs (an enzyme known to be involved in non-homologous end joining repair throughout the cell cycle [52] and in controlling cellular signal transduction following irradiation [53]) were different among these three strains of mouse, *i.e.*, high, immediate, and extremely low activity in the C57BL/6, BALB/cJ, and SCID/J mouse, respectively. Taken together, the data support the hypothesis that a low dose of low LET radiation (as low as 0.05 Gy) is incapable of inducing genomic instability (determined by the presence of late occurring chromosomal damage) in BM cells collected at 6 mo after exposure of mice, regardless of the intrinsic activity of DNA-PKcs in exposed individuals. This information should have value in improving the assessment of low-dose health risk. Hence, the results from our studies are highly relevant to public health due to the fact that there is unavoidable exposure to low-level background radiation and an increased use of low-dose radiation in daily life (e.g., for medical diagnosis or airport safety), as mentioned previously.

We recognize that the level of background radiation or that which is commonly used in medical diagnosis or airport safety is much lower than the 0.05 Gy of ^137^Cs γ rays used in our study. Further, an acute exposure to a single low dose of the 0.05 Gy of ^137^Cs γ rays used in this study does not fully mimic chronic human exposure to low-dose radiation. However, our goal is to use the findings obtained from this study, including those from our previous work [1], as the first step for future investigations on the mechanisms linked to the potentially non-harmful effects of low-dose radiation encountered in the environment or associated with daily life. Hence, to better mimic human exposure to low-dose radiation in daily life and to advance our understanding of mechanisms for possible non-harmful effects associated with exposure to low-dose radiation, further investigations should be conducted to determine the effects of a regimen of exposure to a series of doses (*i.e.*, repeated, or chronic, or fractionated exposure) and of a dose-rate series of the total dose. 

It should be noted that the frequencies of reciprocal translocations (in particular those reciprocal translocations involving two nP chromosomes) or inversions might have been underestimated. This is because whole mouse-genome mFISH, or spectral karyotyping (SKY), or G-banding methods are required in order to accurately score exchanges between two nP chromosomes. None of these more sophisticated cytogenetic methodologies was used in this study. With respect to Robertsonian translocations (RT) or centric fusion occurring between nP chromosomes, it is easy to recognize them without using the whole mouse-genome mFISH, or SKY, or the G-banding method. This is because all normal mouse chromosomes are telocentric. Further, it was impossible to determine whether a clone of cells existed without the use of the whole mouse genome mFISH, or SKY**,** or the G-banding method, unless a clone of a specific type of aberration in a mouse occurred between two of the painted chromosomes in the same mouse. 

The finding of a lack of genomic instability in BM cells of SCID/J mice exposed to 0.05 Gy of ^137^Cs γ rays suggests that BM cells of SCID/J mice acquire other pathways for a repair of radiation-induced DSBs. Further, although apoptosis was not investigated in this study, it is possible that BM cells of SCID/J mice are capable of removing some of the damaged cells by means of apoptosis. The non-harmful effects of low doses and low dose-rates of low LET radiation *via* apoptosis have previously been reported in the spleen of SCID mice [54,55]. In those studies, a role for p53 proteins in apoptosis induced by low dose or low dose-rate of low LET radiation has been suggested. Nevertheless, it is known that cellular responses to DNA damage (caused by either endogenous metabolic processes or exogenous sources, e.g., ionizing radiation and chemicals) constitute a complex system involving the recognition of the induced damage and the initiation of signal transduction cascade(s) [53]. Hence, it is reasonable to speculate that various signaling pathways may be involved in protective effects of low-dose radiation after exposure of SCID/J mice to low-dose radiation. Such signaling pathways may include ataxia telangiectasia-mutated protein, protein kinase C, activator protein 1, mitogen-activated protein kinases, or nuclear factor-kappa B. However, none of these pathways was investigated in this study, making it important to conduct further studies for enhancing our knowledge of the mechanisms associated with the potentially *in vivo* non-harmful effects of low-dose radiation. Such information would provide a basis for a better assessment of health risk from exposure to low-dose radiation.

## 4. Conclusions

Our results indicate no increase in the frequency of late-occurring chromosomal damage in the 0.05-Gy-exposed SCID/J mice at 6 mo post-irradiation. Further, the data are consistent with our previous observations in BABL/cJ mice (containing an intermediate level of endogeneous DNA-PKcs activity) exposed to the same low doses of low-LET radiation. Taken together, the data support the hypothesis of no evidence for *in vivo* induction of genomic instability by low-dose radiation, although the mechanisms involved in such a phenomenon are inadequately understood at the present time, and thorough future investigations are required.
